# Citric Acid Cross-Linked Gelatin-Based Composites with Improved Microhardness

**DOI:** 10.3390/polym16081077

**Published:** 2024-04-12

**Authors:** Abdulrraouf Taboun, Marija Jovanovic, Milos Petrovic, Ivana Stajcic, Ivan Pesic, Dusica B. Stojanovic, Vesna Radojevic

**Affiliations:** 1 Faculty of Technology and Metallurgy, University of Belgrade, Karnegijeva 4, 11120 Belgrade, Serbia; abdurraouftaboun@gmail.com (A.T.); marija.jovanovic@tmf.bg.ac.rs (M.J.); mpetrovic@tmf.bg.ac.rs (M.P.); duca@tmf.bg.ac.rs (D.B.S.); vesnar@tmf.bg.ac.rs (V.R.); 2 Department of Physical Chemistry, “Vinča” Institute of Nuclear Sciences—National Institute of the Republic of Serbia, University of Belgrade, Mike Petrovića Alasa 12–14, P.O. Box 522, 11001 Belgrade, Serbia; 3 Center for Microelectronic Technologies, Institute of Chemistry, Technology and Metallurgy—National Institute of the Republic of Serbia, University of Belgrade, Njegoševa 12, 11000 Belgrade, Serbia; ivan.pesic@ihtm.bg.ac.rs

**Keywords:** gelatin, hybrid composite, calcium phosphates, microindentation

## Abstract

The aim of this study is to investigate the influence of cross-linking and reinforcements in gelatin on the physico-mechanical properties of obtained composites. The gelatin-based composites cross-linked with citric acid (CA) were prepared: gelatin type B (GB) and β-tricalcium phosphate (β-TCP) and novel hybrid composite GB with β-TCP and hydroxyapatite (HAp) particles, and their structure, thermal, and mechanical properties were compared with pure gelatin B samples. FTIR analysis revealed that no chemical interaction between the reinforcements and gelatin matrix was established during the processing of hybrid composites by the solution casting method, proving the particles had no influence on GB cross-linking. The morphological investigation of hybrid composites revealed that cross-linking with CA improved the dispersion of particles, which further led to an increase in mechanical performance. The microindentation test showed that the hardness value was increased by up to 449%, which shows the high potential of β-TCP and HAp particle reinforcement combined with CA as a cross-linking agent. Furthermore, the reduced modulus of elasticity was increased by up to 288%. Results of the MTT assay on L929 cells have revealed that the hybrid composite GB-TCP-HA-CA was not cytotoxic. These results showed that GB cross-linked with CA and reinforced with different calcium phosphates presents a valuable novel material with potential applications in dentistry.

## 1. Introduction

The diverse tissue defects resulting from diseases and trauma have spurred numerous research initiatives aimed at discovering novel composite materials facilitating the regeneration of damaged tissues and organs. Regenerative endodontics seeks to promote the natural healing and regeneration of dental tissues, particularly the pulp–dentin complex, with its vascularization, innervation, and peripheral dentin [1]. Traditional endodontic procedures typically entail completely removing damaged pulp tissues from teeth, followed by disinfection and medication [2]. For an extended period, this radical approach has been perceived as the sole treatment option, devoid of alternative strategies. Composite materials play an important role in advancing regenerative endodontic treatment, a therapeutic approach aimed at restoring damaged or diseased dental pulp, serving as three-dimensional structures that support cell growth, tissue formation, and overall tissue engineering within the root canal system [3]. Gelatin is known for its excellent biocompatibility, making it well-suited for use in medical applications [4]. As a derivative of collagen, a major component of the extracellular matrix, gelatin provides a natural and supportive environment for cells. Its bioactivity promotes cell adhesion, proliferation, and differentiation. Ribeiro et al. [5] developed an injectable antibiotic-laden fibrous microparticle gelatin methacryloyl hydrogel, which showed promise in the ablation of endodontic infections. In a study published by Londero et al. [6], it was found that a gelatin-based scaffold combined with a blood clot improved tissue repair in immature dog teeth undergoing regenerative endodontic treatment. Agarwal et al. [7] demonstrated the potential of gelatin-carboxymethyl chitosan scaffolds for dermal tissue engineering with high porosity, water uptake, and cell adhesion. Aldana [8] reviewed the use of electrospun gelatin-based materials in bone, cartilage, skin, nerve, and ocular and vascular tissue engineering, highlighting their physicochemical and biocompatibility properties.

A range of studies have explored the potential of gelatin-based composites in tissue engineering. Sun et al. [9] found that a gelatin/polyvinyl alcohol composite exhibited improved thermal stability and mechanical properties, with potential applications in high-strength tissue engineering. Lacroix et al. [10] reported that gelatin–bioactive glass composite materials with controlled macroporosity exhibited enhanced mechanical properties and in vitro bone-like apatite-forming ability, suggesting their potential for bone regeneration. Asadpour [11] further expanded the potential of gelatin-based composites by incorporating silk fibroin and chitosan, resulting in scaffolds with suitable porosity, water uptake, and degradation rate, as well as demonstrated biocompatibility. Yan et al. [12] reported that the addition of graphene oxide to gelatin/polyacrylamide hydrogels significantly improved their mechanical properties, including elastic modulus and fractured stress. Waiyawat et al. [13] developed a gelatin-based gel containing calcium phosphate nanoparticles for treating dentine sensitivity. Belonging to the category of bioceramics, calcium phosphates (CaPs) exhibit outstanding biological, physical, and chemical properties owing to their composition, closely resembling that of compounds found in teeth and bones [14,15,16]. This similarity ensures excellent biocompatibility within a host body. Moreover, their capacity to release calcium (Ca) and phosphorus (P) ions, facilitating tooth remineralization, positions CaPs as valuable materials for various applications in dentistry. The most widely recognized bioceramics based on calcium phosphate include hydroxyapatite (HAp) (Ca_10_(PO_4_)_6_(OH)_2_) and tricalcium phosphate (β-TCP) (Ca_3_(PO_4_)_2_) [17]. 

Extensive research has been dedicated to composite materials incorporating HAp alongside natural or synthetic polymers. Various composites, such as those made of nano-hydroxyapatite/polymer, porous chitosan sponge, and a biodegradable composite with an interconnected spherical network, have shown promise in tissue engineering [18,19,20]. These materials offer controllable porosity, good mechanical strength, and improved biological activity, making them suitable for load-bearing bone repair and regeneration. Biological sources of HAp have been employed to create composites, such as gelatin/chitosan/fibrin/bone ash/HAp, showing high biocompatibility and presenting a safe option for bone replacement [21]. Electrospun nanofiber composite materials incorporating insulin-modified HAp/PLGA (poly (lactic acid-co-glycolic acid)) exhibit significant osteogenesis potential, positioning them as promising materials for artificial bone production [22]. Sharma et al. [23] introduced a combination of nano-HAp, gelatin, and acrylic acid for tooth filling. The prepared dental material proved to be non-toxic, which indicates that it could be applied in restorative dentistry. 

Extensive research has also been conducted on the β-TCP, including its incorporation as a bone substitute [24,25,26]. Similar to HAp, β-TCP is not naturally occurring but can be effectively synthesized using high-temperature methods [27]. It exhibits superior thermal stability compared to HA and undergoes faster resorption owing to its solubility. Research on tricalcium phosphate (β-TCP) in dentistry has shown promising results. Park et al. [28] investigated 3D-printed β-TCP-containing PCL composites for dental tissue engineering, revealing improved cell proliferation and alkaline phosphate activity. Roca-Millan [29] highlighted the potential of β-TCP as a synthetic graft material in implant dentistry, with comparable outcomes to other materials. The most recent study by Zamora et al. [17] aimed to innovate materials for endodontic treatments by investigating β-TCP and commercial and natural HAp, in combination with chitosan (CS), in varying proportions, proving the high biocompatibility of all three experimental materials.

Cross-linking is applied to overcome the poor mechanical properties of gelatin. Usually, the cross-linking agents for biomedical uses are glutaraldehyde (GTA) [30,31,32] and genipin [33,34,35]. In their papers about cross-linking by GTA, Lin et al. [30] investigated the influence of pH on the cross-linking process and, hence, on the mechanical properties of gelatin film, while Gu et al. [31] showed the better mechanical properties of gelatin-TCP-HA scaffolds after cross-linking with promoted cell proliferation and osteogenic differentiation. Zhang [32] evaluated the cross-linking process of electrospun gelatin nanofibers with improved mechanical properties and thermostability. In research with genipine cross-linked gelatin, Bigia et al. [33] and Kirchmajer [34] revealed increased tensile compression strength with increased genipin content. In the other work, Solorio [35] processed gelatin microspheres cross-linked by genipin with a controlled release profile of growth factors. Although the presented cross-linking agents show potential, GTA shows some toxicity, while genipin is very expensive, justifying the investigation of more suitable solutions for gelatin cross-linking. Citric acid (CA) is a polyfunctional monomer with high cytocompatibility and solubility. It is a non-toxic product of the body’s metabolic process called Krebs or citric acid cycle, which is a part of cellular respiration [36,37,38,39,40,41]. Also, CA is commercially available at low cost. The cross-linking process is based on the participation of CA in hydrogen bonding in a polymer network [36]. In their research, Uranga et al. [36] and Zhao et al. [39] improved mechanical properties by cross-linking gelatin and collagen, respectively, with CA, while Hasan et al. [37] focused on thermal properties and antioxidant and antimicrobe activities of cross-linked gelatin fibers. On the other hand, Rocha-García et al. [40] have examined the influence of cross-linking on the drug release profile of thermo-sensitive gelatin/poly(ethylene glycol) diamine cross-linked citric acid hydrogels.

However, there is a lack of studies investigating gelatin cross-linked with citric acid (CA) and modified with both HAp and β-TCP and the influence of varying parameters on microhardness, which is crucial in dental restorative materials. This study presents a gelatin–matrix composite cross-linked with CA and reinforced with HAp and β-TCP particles with enhanced microhardness, paving the path for further investigations in the field of dental composite materials with potential as load-bearing structures.

## 2. Materials and Methods

### 2.1. Materials 

Type B gelatin (GB) from bovine skin (∼225 g Bloom), citric acid (CA), hydroxyapatite (HAp), and the β-tricalcium phosphate (TCP) were purchased from Sigma–Aldrich (Burlington, MA, USA). 

### 2.2. Preparation of Samples

The scaffolds were processed using the solution casting method. The composition of prepared samples is given in Table 1. The 10% *w*/*w* solution of GB distilled water was stirred at 50 °C for 2 h. The β-TCP was added to the gelatin solution (5% *w*/*w* relative to GB), and it was stirred until obtaining a homogenous solution. The same procedure was repeated for samples with HAp and hybrid with β-TCP and HAp. The samples with CA were prepared by adding 25% *w*/*w* of CA in a 10% *w*/*w* solution of GB in distilled water. After stirring the solution for 1 h, 5% *w*/*w* (relative to GB) β-TCP/HAp was added and continuously stirred for 2 more hours at 50 °C. Finally, each solution was poured into a separate Petri dish and left to dry for 48 h at room temperature. The samples with CA, for cross-linking reaction, were subjected to heating at 90 °C for 24 h [40]. 

### 2.3. Characterization of Samples

#### 2.3.1. FTIR Spectroscopy

The processing and possible establishment of chemical bonding during processing was followed by FTIR analysis (Fourier-Transfer Infrared Spectroscopy with attenuated total reflectance (ATR) mode). The spectra were recorded by Nicolet 6700 spectrometer (Thermo Scientific, Waltham, MA, USA) in the wavelength range from 2.5 μm to 20 μm (i.e., 4000 cm^−1^ to 500 cm^−1^). 

#### 2.3.2. DSC Analysis

The thermal properties of samples were revealed using DSC analysis by DSC 60 Plus (Shimadzu, Kyoto, Japan). The measurements were performed in the temperature range from 25 °C to 150 °C for series with β-TCP and 180 °C for series with β-TCP and HAp under a dynamic nitrogen flow of 50 mL/min. Samples weighing 5–9 mg were investigated. The samples were heated up at a rate of 10 °C/min. 

#### 2.3.3. FESEM Microscopy 

The morphology and structure of samples were examined by Field Emission Scanning Electron Microscope (FESEM) (TESCAN MIRA 3, Brno, Czech Republic). The surfaces of the samples were sputtered with gold. 

#### 2.3.4. Microindentation

Microindentation is a useful method for evaluating the mechanical properties of biomaterials because it enables the measurement of the dynamic properties of composites under conditions that resemble the ones they will be subjected to during use [41]. Hardness is conventionally defined as the resistance to permanent penetration to an indenter made of a more rigid material, while the measurement is only performed once the load is removed, and elastic deformation is not considered. On the other hand, microindentation allows for the measurement of the indentation depth during the applied load F, so both plastic and elastic deformations that occur during indentation are considered during the test. The microindentation, therefore, determines the contact stiffness and, thus, the indentation modulus Er, as well as the indentation hardness H. This method allows for monitoring the time dependence of material properties, such as the work ratio of elastic and plastic deformation during testing. A prescribed load is also applied to the indenter in contact with the sample. As the load is gradually achieved, the depth of penetration is measured. The impression surface at full load is determined using the penetration depth and the indenter’s known dimensions. Hardness is determined as the ratio of the applied load and the calculated surface of the impression; the modulus of elasticity can be determined from the unloading curve [41].

Mechanical characteristics were determined through a microindentation test on the Texture Analyzer Shimadzu EZ Test LX (Shimadzu, Kyoto, Japan), equipped with a 500 N load cell. The indenter was a ball with a 4 mm diameter, and the device was programmed to perform loading–unloading associated with this type of test [40]. It was set to gradually increase the force at a 0.25 N/s rate until it reached the 5 N load, maintain that load for 20 s, and then gradually unload at the same rate until the full release. 

**Figure 1:**
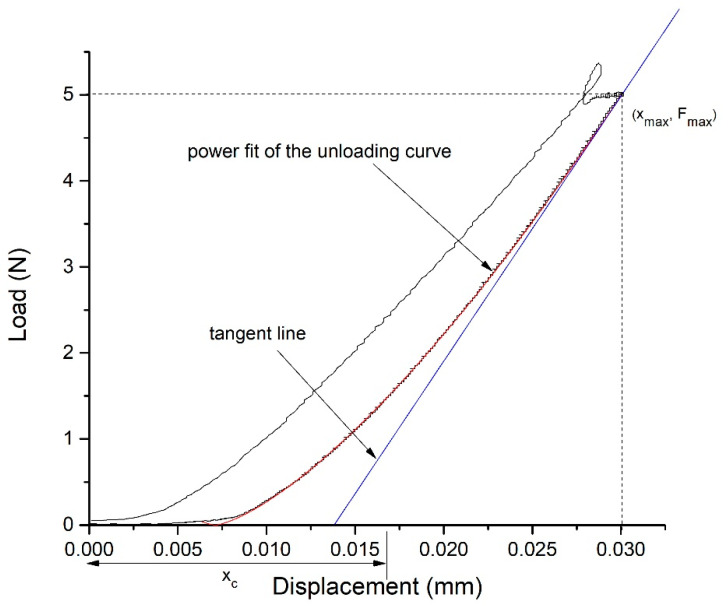
Load vs. displacement diagram: power fit of the unloading curve (red line), tangent line at the maximum load, and displacement (blue line).

The standard output of the test is shown in Figure 1. 

The methodology developed by Oliver–Pharr was used to determine the hardness and the reduced modulus of elasticity of the samples [42]. The unloading segments of the load vs. displacement measurements were used to fit a power function curve of the following form:

(1) $$F=C\cdot {\left(x-x_{f}\right)}^{m},$$

where *F* represents the current load, *x* is the current displacement, and *x_f_*, *m*, and *C* are the parameters of the fit. The stiffness was calculated as the slope of the tangent line on the unloading curve at the point of the maximum displacement and load (*x*_max_, *F*_max_), i.e.,

(2) $$S={\left.\frac{dF}{dx}\right|}_{x=x_{max}},$$

and the contact depth was calculated as the following:

(3) $$x_{c}=x_{max}-\varepsilon \cdot \frac{F_{max}}{S}$$

where *ε* = 0.75, since the spherical indenter is of a paraboloid of revolution type.

The hardness of the sample, *H*, was determined based on the projected area of the hardness impression, *A*, of a spherical indenter with a diameter *d*:

(4) $$A=\left(d\cdot x_{c}-x_{c}^{2}\right)\cdot \pi$$

(5) $$H=\frac{F_{max}}{A}$$

Additionally, the reduced modulus of elasticity, *E*_r_, was calculated as the following:

(6) $$E_{r}=\frac{S}{2}\cdot \sqrt{\frac{\pi }{A}}$$

The two-way ANOVA statistical analysis was performed in Origin 9.0 software (OriginLab Corporation, Northampton, MA, USA) to determine the statistical significance of the obtained results from the microhardness test.

#### 2.3.5. Cytotoxicity Assay 

The cytotoxicity of the composites was performed according to ISO 10993-5 [43] using an MTT assay. The principle of the cytotoxicity test is based on testing the effect of material extracts, i.e., materials in liquid form in indirect contact with target cells, L929, and cultivated media. Based on the appearance and viability of the examined cells after culture in direct contact with the test material (direct test) or the metabolic activity of the cells in indirect contact (indirect test), the degree of cytotoxicity is assessed and expressed in the form of indices and/or numerical values.

Briefly, L929 cells were cultivated in flat-bottom 96-well microtiter plates (Thermo Fisher Scientific, Waltham, MA, USA), in complete cultivation media—RPMI 1640 supplemented with 10% FCS and penicillin/streptomycin (all from Capricorn, Ebsdorfergrund, Germany), starting in density 1 × 10^4^ cells/well. When they become semi-confluent (70–80% of confluency), the treatment was added on cells instead of cultivation medium. Treatment was prepared as conditioned media—test sample extracts from solid material (GB-TCP-CA and GB-TCP-CA-HA) and negative control (aluminum oxide stick) were prepared by placing the material samples in sterile polypropylene test tubes in the complete RPMI medium (the ratio of the surface area of the material to the volume of the medium should be 3 cm^2^/mL) for 24 h. Conditioned medium was used in an indirect assay at different dilutions (100%, 50%, 25%) in the complete medium. As a positive control, the 5% phenol solution (Merck) was used. Each dilution of conditioned medium, as well as negative and positive control, was tested in 6 wells with L929 cells. After 24 h, 3-(4,5-Dimethylthiazol-2-yl)-2,5-Diphenyltetrazolium Bromide (MTT) was added on cells in concentration of 0.5 mg/mL. After 1 h of incubation on 37 °C, the reaction was stopped with Dimethyl Sulfoxide (both from Thermo Fisher Scientific). 

The assessment of cytotoxicity based on the metabolic activity of the cells in the indirect test is evaluated based on the reaction of the sample with the MTT test. The absorbance of the solution is read at 570 nm wavelength, subtracting the reference wavelength at 690 nm (optical correction) (OD570 = abs570 − abs690). The OD of 570 in untreated cells is a measure of exponential growth of 1 × 10^4^ cells seeded 24 h before the assay. Blank controls are additionally used in the test, i.e., solutions of the substance alone and MTT, without cells present (OD570 blank). Cell viability is calculated based on the following formula:

$$Viability\;(\%)=\frac{100\times ({OD}_{570}\;sample-\Sigma {OD}_{570}\;blank/6)}{\left(\Sigma {OD}_{570}\;control/6-\Sigma {OD}_{570}\;blank/6\right)}$$

To show a cytotoxic effect, a measure of 30% reduction in viability compared to the control for 100% extract is taken. Range of reference values: 0—absence of cytotoxicity (0–30% decrease in viability compared to control (100%)); 1—discrete cytotoxicity (30–50% decrease in viability compared to control); 2—moderate cytotoxicity 50–75% decrease in viability compared to control); 3—pronounced cytotoxicity (75–100% decrease in viability compared to the control).

In order to stain the treated cells with Hematoxylin and confirm results obtained by MTT test, L929 cells were cultivated on cover slips in 24-well plates (Thermo Fisher Scientific), in a density of 5 × 10^4^ cell/well. When they become semi-confluent (70–80% of confluency), the treatment was added on cells instead of cultivation medium in the same manner as described above. After 24 h treatment, the cells on cover slides were washed in phosphate buffer saline (PBS), pH 6.5, and stained in Mayer Hematoxylin (Merck, Rahway, NJ, USA) for 90 s, upon which they were washed in tap water. Cover slides were mounted with mounting medium on microscope slides and analyses were performed on light microscope (Carl Zeiss, Axio Imager, Oberkochen, Baden-Württemberg, Germany).

## 3. Results and Discussion

### 3.1. FTIR Analysis

**Figure 2:**
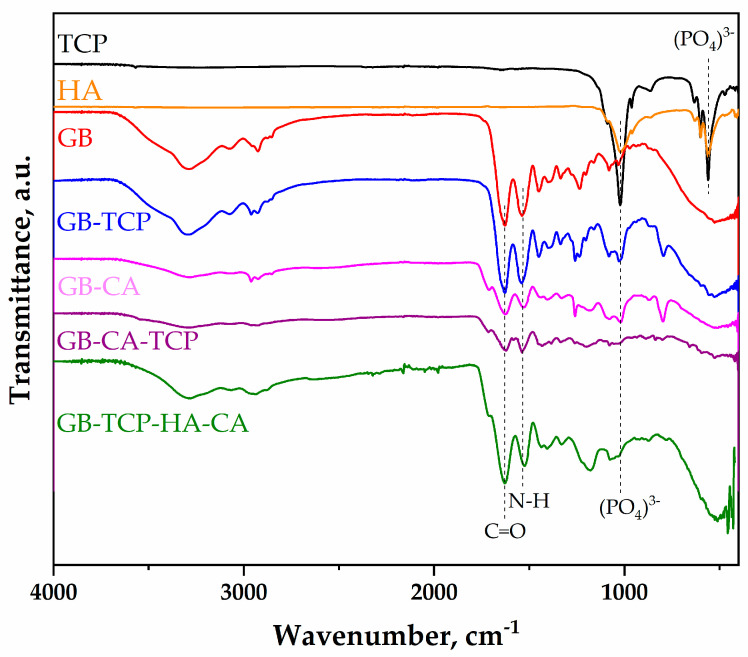
FTIR spectra of starting particles: the β-tricalcium phosphate (TCP) and hydroxyapatite (HAp) solution-casted films: GB and GB-TCP and thermally treated samples with CA: GB-CA, GB-CA-TCP, and GB-TCP-HA-CA.

The FTIR analysis was conducted with the aim of identifying the possible new chemical bonding established in the scaffold during processing. FTIR spectra of starting materials (GB, β-TCP, and HAp) and the obtained films (GB-CA, GB-TCP, GB-TCP-CA, GB-TCP-HA-CA) are presented in Figure 2. Characteristic peaks and functional groups for GB are present in all scaffolds. Embedding of β-TCP and HAp in the GB matrix has not changed the characteristic peaks. The characteristic peaks in both β-TCP and HAp were located at 970 and 1021 cm^−1^, attributed to the stress vibrational modes of the (PO_4_)^3−^ bond and vibration peaks at 600 and 540 cm^−1^ of the (PO_4_)^3−^ [17]. The Amide I peak of GB corresponding to the vibration of the amide carbonyl group was shifted due to the cross-linking process from 1629 cm^−1^ to 1624 cm^−1^, 1621 cm^−1^, and 1619 cm^−1^ for GB-CA, GB-TCP-CA, and GB-TCP-HA-CA, respectively [44]. Also, the cross-linking process has shifted the Amide II band attributed to the bending of the N-H bond from 1541 cm^−1^ to 1528 cm^−1^, 1523 cm^−1^, and 1528 cm^−1^ for GB-CA, GB-TCP-CA, and GB-TCP-HA-CA, respectively [45,46]. Based on these findings, it can be concluded that the helix structure of gelatin was altered due to a cross-linking process [47,48,49]. Furthermore, cross-linking was not disrupted by the agglomerated β-TCP and HAp particles, which is important for further thermal and mechanical performance evaluation.

### 3.2. DSC Analysis

**Figure 3:**
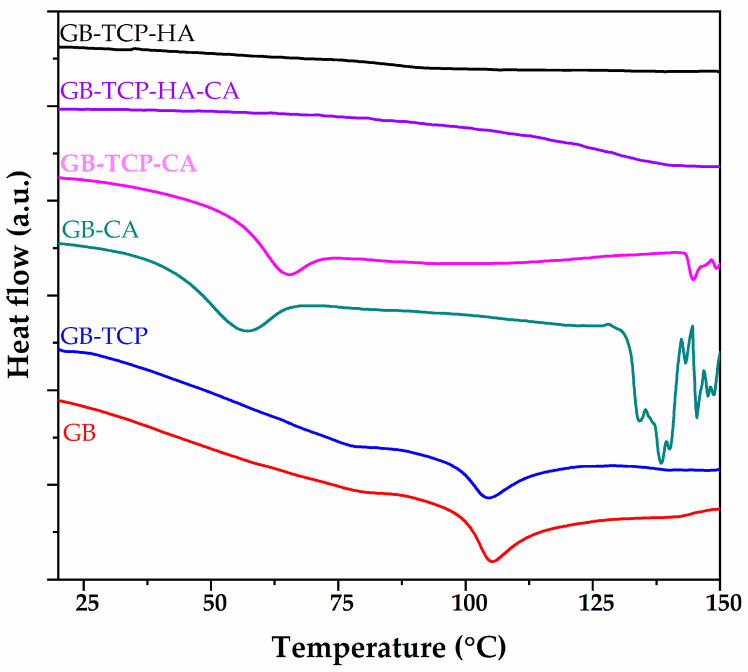
DSC analysis of matrix and composite materials.

The results of DSC analysis—the T_g_ and T_m_ of samples—are presented in Figure 3. It can be seen that samples have shown a semi-crystalline structure due to the presence of both T_g_ and T_m_. The reduction in T_g_ value for sample GB-TCP-HA compared to pure GB could be the consequence of the formation of large agglomerates that act as plasticizers. However, as the temperature increases, thermally stable agglomerates present obstacles in a phase transition, increasing T_m_ for hybrid composite GB-TCP-HA in comparison with pure GB. After adding citric acid and thermal treatment, it looks like the crystallinity of the polymer is increased. However, together with the decrease in the melt temperature, it could be concluded that cross-linking occurred. The crystalline structure of gelatin mostly results from its α-helix and triple helical structure. During cross-linking, the carboxylate groups in CA react with the gelatin’s hydroxyl and amine groups, leading to lower crystallinity. It can also be noticed that the isomerization of the peptide bonds in gelatin from the trans to the cis configuration occurred in samples GB-CA and GB-CA-TCP from 130 °C and 143 °C, respectively [50,51,52,53]. This could be a consequence of lower helical conformation due to the cross-linking process [54]. 

### 3.3. Mechanical Properties

**Figure 4:**
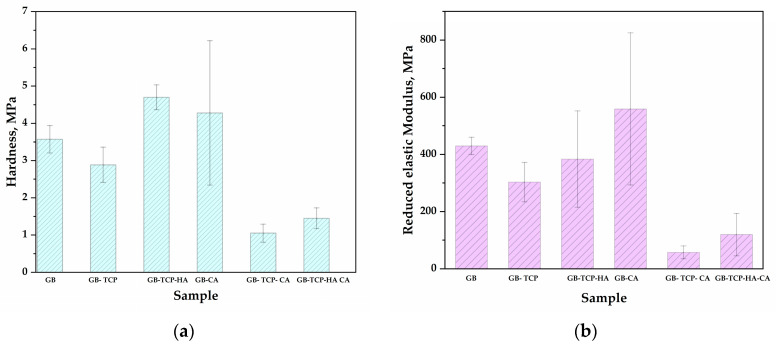
Results of microindentation test (mean values with standard deviations): (**a**) hardness; (**b**) reduced modulus of elasticity.

Results of the microindentation test are presented in Figure 4a,b as mean values obtained for hardness (H) and reduced modulus of elasticity (E_r_), with standard deviation. Embedding particles in the polymer matrix leads to an increase in both H and E_r_ values. Looking at the mean values, the addition of β-TCP improved the E_r_ by 149%, while the hybrid composite had a 60% increase in E_r_; hardness was increased by 42% and 118%, respectively. Cross-linking leads to further improvement of mechanical properties. The hardness of GB-CA, GB-TCP-CA, and GB-TCP-HA-CA was 49%, 449%, and 320% higher, respectively, compared to non-crosslinked samples, while Er increased by 30%, 288%, and 122%, respectively. 

The ANOVA analysis of the influence of particle addition and the cross-linking process on the mechanical properties was performed. The factors that were used for the analysis comprised the addition of HAp, β-TCP particles, or both (F1) and the use of the CA as a cross-linking agent (F2) in the tested samples. The analysis has shown that both F1 and F2, as well as their interaction, significantly affect the hardness of the tested samples (at the confidence level of *p* < 0.01). According to the Tukey post-hoc test [44], there is a statistical difference between the hardness of the samples that contain β-TCP or both β-TCP and HAp compared to the samples without any particles (*p* < 0.01). Regarding the reduced elasticity modulus, the ANOVA analysis has shown that both F1 (*p* < 0.01) and F2 (*p* < 0.05) significantly affect it. The Tukey post-hoc test identifies that there is a statistical difference between the reduced elasticity modulus of the samples that contain β-TCP and both β-TCP and HAp (*p* < 0.05) and even higher statistical difference between samples that contain β-TCP compared to the samples without any particles (*p* < 0.01).

Both hardness and E_r_ for GB-TCP-HA-CA were lower than for GB-TCP-CA but still significantly higher than for GB. This reduction in comparison to GB-TCP-CA could be explained by the agglomeration of hybrid particles and the interaction between HAp and β-TCP [17]. Also, DSC analysis revealed that the lower T_g_ for GB-TCP-HA-CA indicated that the cross-linking was affected by the presence of hybrid reinforcements.

### 3.4. FESEM Analysis of Matrix and Composites

**Figure 5:**
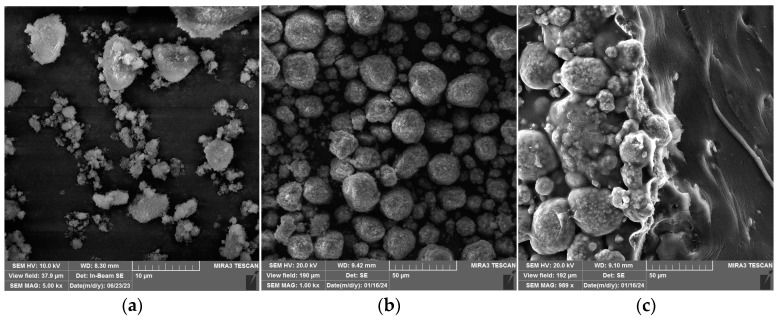

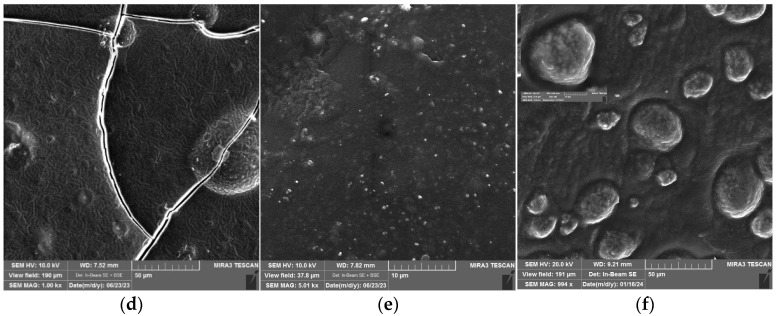
FESEM images of (**a**) β-TCP (**b**) HAp; (**c**) GB-TCP-HA; (**d**) GB-CA; (**e**) GB-TCP-CA; (**f**) GB-TCP-HA-CA.

The morphology of β-TCP and HAp particles and obtained samples was investigated with FESEM; images are presented in Figure 5. The β-TCP particles with lower dimensions are likely to disperse better and have a better spatial arrangement than larger HAp particles in hybrid composites. In addition, the HAp particles of larger dimensions could serve as stress concentration points and result in declined mechanical properties, i.e., hardness and reduced modulus of elasticity.

**Figure 6:**
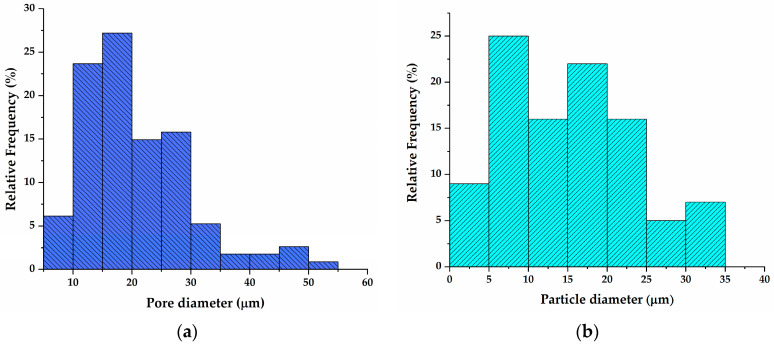
Distribution of: (**a**) pore diameters in GB-CA; (**b**) particle diameter in GB-TCP-HA-CA.

As can be seen from Figure 5d,f, pores present in GB-CA have disappeared with the addition of TCP and HA particles. The distribution of pores in GB-CA is presented in Figure 6a, showing a broad range of pore diameters. On the other side, GB-TCP-HA-CA showed a wide range of particle diameters, which is the consequence of the differences in TCP and HA particle sizes.

### 3.5. Cytotoxicity Assay

**Figure 7:**
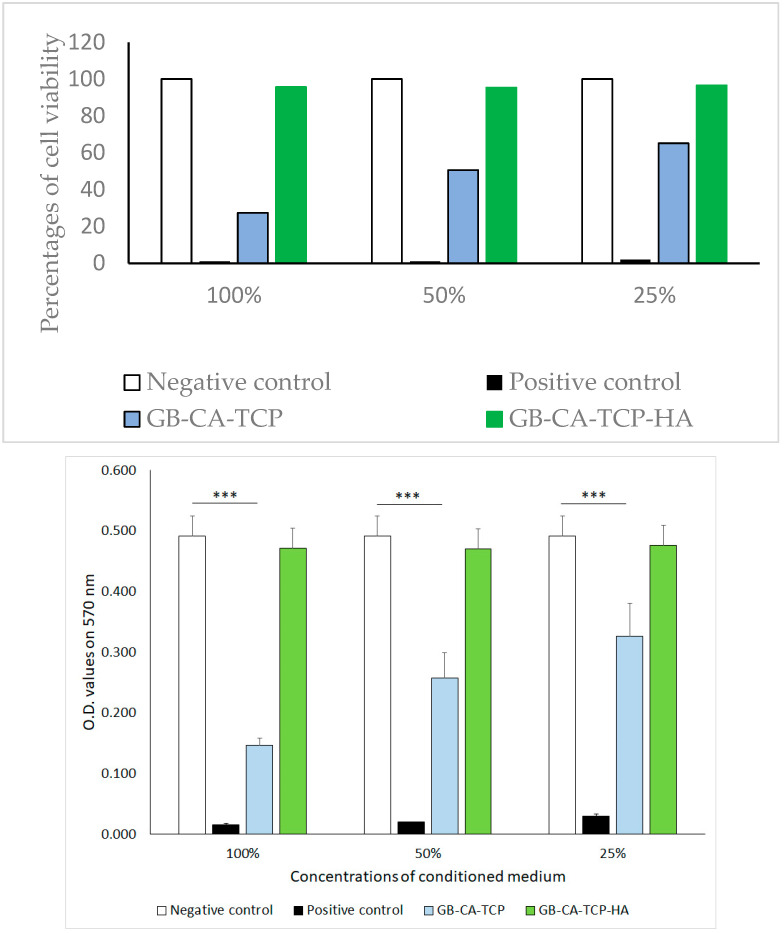
Cytotoxic effect of media conditioned with GB-CA-TCP and GB-CA-TCP-HA on L929 cells based on metabolic activity of cells in an indirect assay (MTT) presented as optical density (O.D.) values obtained on 570 nm. Data are presented as mean ± SD for hexaplicates of treated cells in the culture.(*** represent statistical significance, *p* < 0.001).

An indirect assay for estimation of the cytotoxic effect of the materials (MTT) was performed—the percentages of cell viability are presented in Figure 7 and Table 2.

The percentages of cell viability presented on the graph were calculated using the average values of obtained optical densities at 570 nm of different dilutions of conditioned media and negative control, each in hexaplicates. 

Percentages of cell viability presented in Table 2 were calculated using the average values of obtained optical densities at 570 nm of different dilutions of conditioned media and negative control, each in hexaplicates. The index of cytotoxicity was defined according to the following reference range: 0—absence of cytotoxicity (0–30% decrease in viability compared to control (100%)); 1—discrete cytotoxicity (30–50% decrease in viability compared to control); 2—moderate cytotoxicity 50–75% decrease in viability compared to control); 3—pronounced cytotoxicity (75–100% decrease in viability compared to the control).

**Figure 8:**
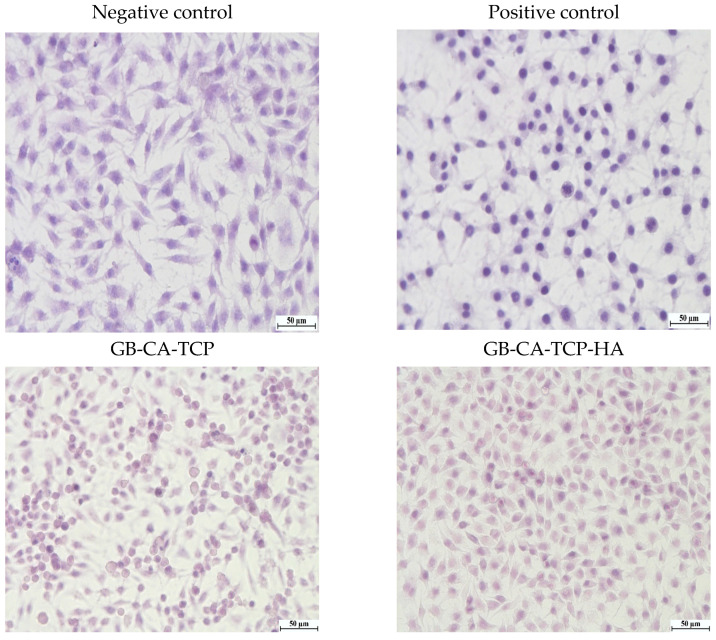
Hematoxylin staining of cells in culture, magnification 40×.

The results of the MTT assay in Figure 7 show that the method used to cross-link gelatin with CA had minor cytotoxicity in composite with TCP, while hybrid composite with TCP and HA has shown no cytotoxicity [55]. That could be explained by the different sizes and spatial distributions of particles in GB-CA-TCP and GB-TCP-HA-CA, which lead to differences in the cross-linking process and content of free CA in composites [56]. Hematoxylin staining of cells treated with conditioned media mirrored the results obtained in the indirect assay (Figure 8). Cells treated with GB-CA-TCP-conditioned media showed the phenotype similar to the one observed in the positive control—most of the cells were in the terminal phase of apoptosis, some of them with condensated chromatin and changed phenotype. On the other hand, the phenotype of cells treated with GB-CA-TCP-HA-conditioned media matched with cells in the negative control—alive and healthy with a few cells in senescence. Hence, these results confirmed the cytotoxicity of cross-linked gelatin with CA in composite with TCP and the absence of a cytotoxic effect of the hybrid composite with TCP and HA.

## 4. Conclusions

The aim of this work was the processing of a composite material based on gelatin with improved mechanical properties, with the use of citric acid as a biocompatible cross-linking agent for the gelatin matrix. The reinforcements for gelatin B were β-TCP and HAp particles, which were chosen as good candidates for dental or endodontic regeneration treatment because of their known biocompatibility and bioactivity. In addition, both calcium phosphates are thermally stable after cross-linking by citric acid at a higher temperature. FTIR analysis has shown that the cross-linking process was successfully performed. The DSC analysis and microindentation revealed improvements in the thermal and mechanical behavior of the obtained composite. The microhardness test was used to follow the mechanical properties as a consequence of cross-linking and reinforcements. Besides the cross-linking process, the reinforcement with β-TCP and HAp also increased T_g_, as well as the hardness and reduced modulus of elasticity. Hardness was increased by up to 449%, while reduced modulus improved by up to 288%. The absence of cytotoxicity in the hybrid composite GB-CA-TCP-HA was revealed by MTT assay on L929 cells and confirmed by Hematoxylin staining of treated cells. Further biocompatibility studies on osteoblastic cells should be performed in future research in order to evaluate the materials’ ability to promote bone growth and tissue formation. The presented results show the promising potential of gelatin-based composites with calcium phosphates stabilized by citric acid. 

## Figures and Tables

**Table 1:** The composition of prepared samples.

Sample	Gelatin B, g	β-TCP, g	HAp, g	CA, g
GB	3	0	0	0
GB-TCP	3	0.15	0	0
GB-CA	3	0	0	1
GB-CA-TCP	3	0.15	0	1
GB-TCP-HA	3	0	0.15	0
GB-TCP-HA-CA	3	0.075	0.075	1

**Table 2:** Viability of cells upon treatment with GB-CA-TCP- and GB-CA-TCP-HA-conditioned media on L929 cells based on metabolic activity of cells in an indirect assay (MTT).

Treatment	Negative Control		Positive Control		GB-CA-TCP		GB-CA-TCP-HA	
	Cell Viability (%)	Index of Cytotoxicity	Cell Viability (%)	Index of Cytotoxicity	Cell Viability (%)	Index of Cytotoxicity	Cell Viability (%)	Index of Cytotoxicity
100%	100	0	1	3	27.3	2	95.3	0
50%	100	0	1	3	50.6	2	95.7	0
25%	100	0	2	3	65.1	1	96.6	0

## Data Availability

The data presented in this study are available on request from the corresponding author. The data are not publicly available due to privacy.
